# Effect of Calcination Temperature on the Phase Composition, Morphology, and Thermal Properties of ZrO_2_ and Al_2_O_3_ Modified with APTES (3-aminopropyltriethoxysilane)

**DOI:** 10.3390/ma14216651

**Published:** 2021-11-04

**Authors:** Damian S. Nakonieczny, Frank Kern, Lukas Dufner, Agnieszka Dubiel, Magdalena Antonowicz, Krzysztof Matus

**Affiliations:** 1 Institute for Manufacturing Technologies of Ceramic Components and Composites, University of Stuttgart, 70569 Stuttgart, Germany; Frank.Kern@ifkb.uni-stuttgart.de (F.K.); Lukas.Dufner@ifkb.uni-stuttgart.de (L.D.); 2 Department of Biomaterials and Medical Devices Engineering, Faculty of Biomedical Engineering, Silesian University of Technology, Roosevelta 40 St., 41-800 Zabrze, Poland; Agnieszka.Dubiel@polsl.pl (A.D.); Magdalena.Antonowicz@polsl.pl (M.A.); 3 Department of Mechanical Engineering, Silesian University of Technology, Akademicka 2A, 44-100 Gliwice, Poland; Krzysztof.Matus@polsl.pl

**Keywords:** zirconia, alumina, 3-aminopropyltriethoxysilane, thermal properties, surface modification

## Abstract

This paper describes the effect of calcination temperature on the phase composition, chemical composition, and morphology of ZrO_2_ and Al_2_O_3_ powders modified with 3-aminopropyltriethoxysilane (APTES). Both ceramic powders were modified by etching in piranha solution, neutralization in ammonia water, reaction with APTES, ultrasonication, and finally calcination at 250, 350, or 450 °C. The obtained modified powders were characterized using X-ray diffraction (XRD), Fourier transform infrared (FTIR) spectroscopy, particle size distribution (PSD), scanning electron microscopy with energy-dispersive X-ray spectroscopy (SEM/EDS), and thermogravimetric analysis (TGA).

## 1. Introduction

Current biomaterials research seeks to identify alternative materials to metals and ceramics, especially for prosthetic and orthopaedic applications [1,2]. Much attention has been paid to polymer–ceramic composites and possible surface modification methods of ceramic fillers to increase their durability [3,4]. The most commonly used fillers for polymer–ceramic composites include ZrO_2_, Al_2_O_3_, SiO_2_, silica microspheres with varying degrees of porosity, HAp, zeolites, and graphene oxide (GO) [5,6,7,8,9]. Aluminosilicate cenospheres are also interesting alternatives to the commonly used oxide and nitride ceramics [10,11]. The main problem encountered with polymer–ceramic composites is ensuring adequate adhesion of ceramic fillers to the polymer matrix [12,13]. One possible method to ensure this is to modify the ceramic surface by introducing functional groups that improve adhesion, e.g., -SH, -CN-COOH, -NH_2_, and -SiH_4_ [14]. Due to the simplicity of applications and the possibility to obtain good adhesion between ceramics and polymers, silanization has received much attention [14,15,16,17]. Silanization is a proven way to introduce silane coupling agents that can be used to improve the surface properties and introduce reactive functional groups on ceramic surfaces [18,19]. It is used to modify the surfaces of different fillers, e.g., carbon nanotubes and those of plant origin [18,19]. Silanization improves the dispersion and interfacial adhesion in the polymer matrix. The organofunctional groups of silanization coupling agents (SCA) can react with the polymer matrix. The hydrolysis of organofunctional groups leads to the formation of a trisilanol group, which can react with the hydroxyl groups on the surface of fillers [18]. SCAs are introduced to modify the usually highly polar ceramic surface, which causes ceramic fillers to aggregate in most hydrophobic polymers [20]. This poor dispersion of fillers causes degradation of the mechanical and chemical properties of polymer–ceramic composites [20]. During silanization, SCAs are often introduced, which are silicon-based chemicals with the capability to form a durable covalent bond between an inorganic material and an organic molecule. The general chemical formula of an SCA is R-(CH_2_)_n_-Si-X_3_, where X is a hydrolysable group, such as methoxy or ethoxy, and R is an organofunctional group, including amino, carboxyl, epoxy, etc. [20]. Many studies of silane coupling agents can be found, i.e., 3-aminopropyltriethoxysilane (APTES) [21], 3-(aminopropyl) trimethoxysilane (APTMS) [22], trimethylchlorosilane (TMCS) [23], (3-mercaptopropyl) trimethoxysilane (MPTMS) [14], tetraethyl orthosilicate (TEOS) [20], triethylenetetramine (TETA) [20], and 3-glycidoxypropyl methyldiethoxysilane (GPTMS) [20]. The selection of an appropriate SCA improves their dispersion in hydrophobic solvents or polymers, resulting in enhanced mechanical properties in polymer composite materials, including epoxy resins [20]. Silanization of oxide ceramics, however, poses a challenge due to its chemical passivity. It is not as uncomplicated as in the case of other materials, i.e., lignins, where direct silanization can be applied and satisfactory results can be obtained [24]. Other studies, e.g., by Caravaca et al., confirm this information and data on surface pretreatment before the actual silanization can be found there [25]. The authors used oxygen plasma to clean the zirconia surface and promote hydroxylation of the surface and improve adhesion for the silane groups from 3-aminopropyldimethylethoxy silane (APDMES) [25]. In the available data, there is some information on the use of compounds and processes such as hydrogen peroxide, ultrasound-assisted sol-gel process with post-treatment freeze-drying, and laser texturing [26,27,28]. All these complementary treatments reduce the number of hydroxyl groups on the surface of modified fillers, increasing their hydrophobicity and improving their dispersion in polymer matrices and resins. This limits the hydrophilicity of the filler and increases their reactivity or compatibility with selected polymers, depending on the type of silane functional group [26]. Additional processes are designed to increase their wettability by the polymer matrix [26]. Nguyen et al. reported that laser texturing using femtosecond, picosecond, and nanosecond laser beams resulted in fillers with bio-inspired hierarchical structures on various materials to produce superhydrophobicity [26]. Additional treatment processes after silanization can introduce super water-repellency [26].

In this study, we carried out a two-stage surface modification process involving alumina and zirconia ceramic fillers. The main objective of our research was to obtain additional surface development on ceramics and to introduce Si- and N-containing compounds on the ceramic surface. As a result, in further applications we could obtain a solid bonding with a polymer matrix, in which the modified powders would constitute ceramic fillers. Such a polymer–ceramic composite would have potential biomedical applications in dental prosthetics and orthopedics. To obtain a high surface development of both types of fillers in the first stage of the process, chemical etching was carried out using fresh, hot piranha solution under reflux with continual stirring. In the second stage, both fillers were silanized with 3-aminopropyltriethoxysilane (APTES) as a 10% solution in 2-propanol. The aim of the two-step etching process was to prepare the surfaces of the ceramic fillers to bond with the functional groups from the APTES and to positively influence the surface development of these powders. Carrying out the calcination process at different temperatures was intended to verify a sufficient temperature range to eliminate organic compounds from the thermal decomposition of APTES. The obtained ceramic powders were then calcined in a muffle furnace at 250, 350, or 450 °C in an air atmosphere. The obtained fillers were characterized to determine the effect of the calcination temperature on their phase composition, characteristic functional groups (especially -NH_2_), thermal properties, and the influence of the two-step modification process on the particle size distribution (PSD).

## 2. Materials and Methods

### 2.1. Sample Preparation

Both ceramic powders were used without prior modification. Zirconia (unstabilized monoclinic, average measured PSD value: 25 μm, Acros Organics, 98.5%, CAS 1314-23-4) and alumina (α-alumina, average measured PSD value: 100 μm, Acros Organics, 99.7%, CAS 1344-28-1) were used. In the first stage, the powders were etched in fresh, hot piranha solution, which was prepared from H_2_SO_4_ (CAS: 7664-93-9, 95%, AVANTOR, Gliwice, Poland) and H_2_O_2_ (CAS: 7722-84-1, 30%, STANLAB) in a volumetric ratio of 3:1. The powders were poured together with piranha solution into a round-bottomed flask and heated under reflux (temperature: 100 °C, time: 15 min) with continuous stirring with a mechanical stirrer in a heating bowl (350 rpm). Then, both powders were filtered under vacuum with a water pump (washed twice with 500 mL). Then, a suspension of both powders in demineralized water was prepared, the pH was measured, and the powders were neutralized with ammonia water (CAS 1336-21-6, 25%, STANLAB, Lublin, Poland) with continuous stirring on a magnetic stirrer (200 rpm). In the second modification stage, both fillers were etched with 3-aminopropyltriethoxysilane (APTES) (CAS: 919-30-2, Acros Organics, Geel, Belgium). For this purpose, a Cp = 10% solution of APTES in 2-propanol (PrOH) (CAS: 67-63-0, Acros Organics) was prepared, into which both powders were added separately. Both powders and the APTES–PrOH solution were stirred on a magnetic stirrer (temperature: 30 °C, time: 24 h, 250 rpm). After the mixing was complete, both suspensions were exposed to ultrasound (*f* = 37 kHz, power: 120%, time: 50 min, temperature: 30 °C, degas mode). Subsequently, zirconia and alumina were filtered under reduced pressure with a water pump (2 × 500 mL wash). The powders were then dried (forced air dryer, time: 12 h, temperature: 80 °C) and calcined in a muffle furnace (RENFERT Magma, Hilzingen, Germany) at temperatures of 250, 350, or 450 °C in an air atmosphere (temperature gradient: 9 °/min, isothermal holding time: 2 h, cooling together with the furnace). The prepared samples were ground in an agate mortar and pestle. The names of the samples are shown in Table 1.

### 2.2. Methods

#### 2.2.1. X-ray Diffraction (XRD)

The crystal structure of the samples was determined by XRD in the 2θ range of 20–70° (X’Pert MPD, PANalytical, Worcestershire, UK) CuKα1, Ge-monochromator, accelerator detector).

#### 2.2.2. Scanning Electron Microscopy (SEM) with Energy-Dispersive X-ray Spectroscopy

(EDS) To confirm the microstructure of ceramic powders, SEM studies were performed using a Zeiss Supra 35 scanning electron microscope (ZEISS, Jena, Germany) with a field emission gun equipped with an UltraDry EDS Detector and Thermo Scientific™ Pathfinder™ X-ray Microanalysis Software. Secondary electron imaging and EDS were used for sample observations at an accelerating voltage of 15 kV and a maximum magnification of 10,000×. Microanalysis Software determined the chemical composition of the analyzed samples. The samples were applied to a conductive carbon tape attached to a sample holder. Powders were not sputtered.

#### 2.2.3. Particle Size Distribution (PSD)

PSD was measured using a Mastersizer 3000 laser granulometer with a Hydro LV wet sample dispersion unit (Malvern Panalytical, Malvern, UK). The modified ceramic powders were dispersed in distilled water in a 50/50 wt.% suspension, and then the dispersant DOLAPIX CE64 (Zschimmer & Schwarz, Lahnstein, Germany) was added to be equal to 0.5 wt.% of the overall weight. The mixture was added into the water-based measuring cell until the obscuration level reached about 10%. To determine the PSD, a Fraunhofer model was used.

#### 2.2.4. Fourier Transform Infrared (FTIR)

FTIR spectroscopy was used to analyze the characteristic functional groups of the ceramic powders. A Shimadzu IR Tracer-100 spectrometer (Michelson interferometer, beam splitter: KBr germanium coated, light source: high-energy ceramics, detector: DLATGS, Kioto, Japan) was used with a multi-reflection ATR attachment equipped with a diamond prism. Transmission spectra were recorded and automatically analyzed with LabSolution IR software. The research was carried out in the medium infrared (mid-IR) range of 4000–400 cm^−1^.

#### 2.2.5. Thermogravimetric Analysis (TGA)

TGA measurements from powders samples were conducted using Q 1500D (MOM, Budapest, Hungary) in nitrogen with a heating rate of 10° min^−1^ in the range from 24 to 1000 °C. Thermal analysis was used to determine the characteristic energetic effects accompanying the thermal decomposition of the substance during heating.

## 3. Results

**Figure 1:**
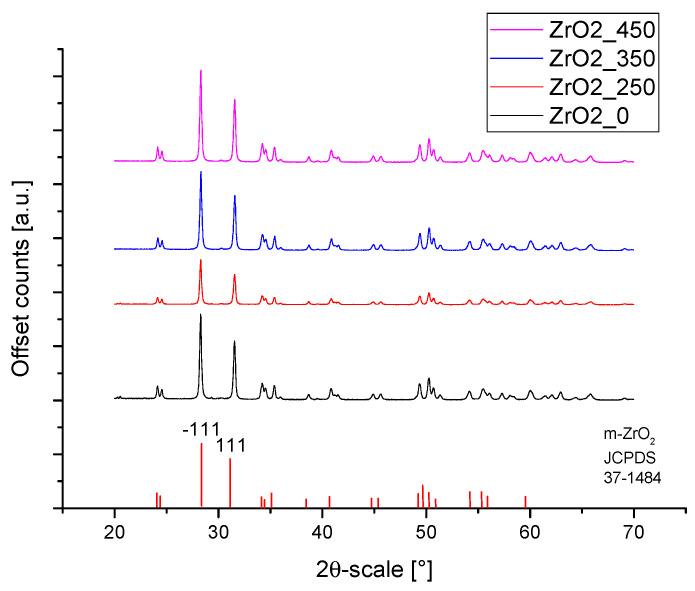
XRD patterns of heat-treated and APTES surface modified zirconia.

Figure 1 shows the XRD patterns of APTES-coated zirconia powders calcined at different temperatures. All powders showed a typical pattern of monoclinic zirconia (P P21/c, JCPDS card no. 01-037-1484). Heat treatment has no apparent effect on the phase composition determined by XRD.

**Figure 2:**
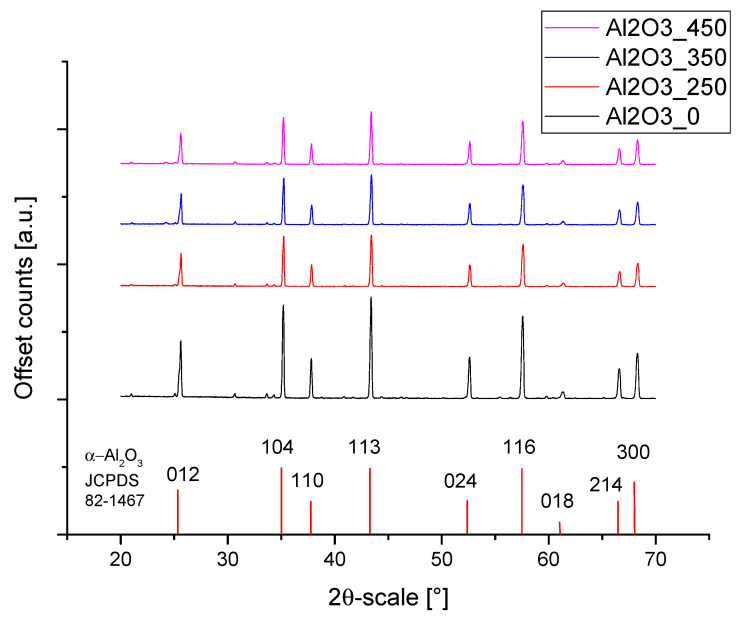
XRD patterns of heat-treated and APTES surface modified alumina.

**Figure 3:**
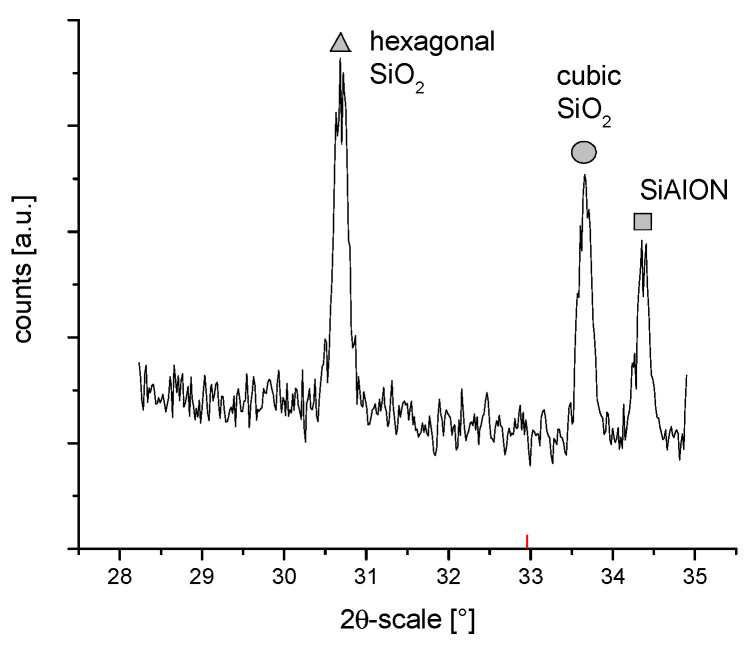
Magnified detail XRD pattern of alumina sample—Al_2_O_3__450 between 29–35°.

Figure 2 shows the XRD patterns of the coated alumina powders. All powders showed a typical pattern of α-alumina as the main constituent (rhombohedral, R-3c, JCPDS card 01-082-1467). There were, however, some smaller peaks between 2θ = 28–35° which indicate other components such as different types of silica: hexagonal, P3121, JCPDS card 01-080-2148 at 30.7°, cubic Fm3m (JCPDS-01-089-3609 at 33.6°), and possibly a hexagonal aluminium-silicon-oxynitride phase (JCPDS 00-48-1618 at 34.4°) (Figure 3). The accurate identification of these minor constituents remains elusive, as only the most pronounced peaks were visible and minor reflections were at the noise level.

However, this observation is in good accordance with the EDS data shown in the following section. These silicon-containing compounds, which are likely the decomposition products of APTES, are probably also present in the zirconia samples (except for the SiAlON compound), but this cannot be verified due to the dominance of the zirconia peaks.

Microscopic observations were carried out for all samples: raw powders (blind samples), modified (non-calcined ZrO2_0 and Al2O3_0), and calcined at 250, 350, and 450 °C. Table 2 shows the SEM-EDS chemical composition measurements, which were derived from averaging four measurements.

**Figure 4:**
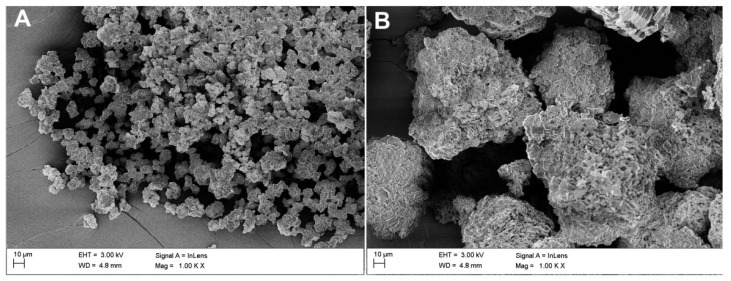
Starting powders: (**A**) raw ZrO_2_ and (**B**) raw Al_2_O_3_.

The pictures below show the morphology of the raw powders (Figure 4). Based on SEM observations, it was found that the untreated ceramic powders tended to clump together in agglomerates.

**Figure 5:**
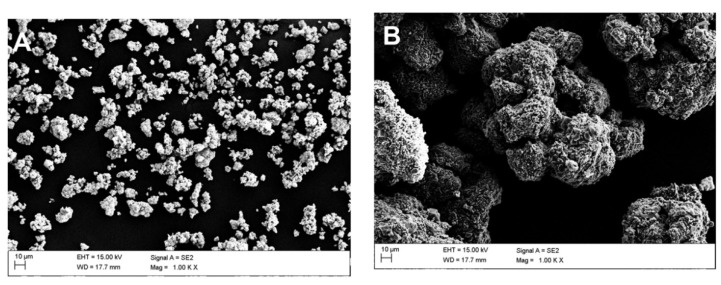
Morphology of the APTES-modified zirconia and alumina powders: (**A**) ZrO_2__0 and (**B**) Al_2_O_3__0.

**Figure 6:**
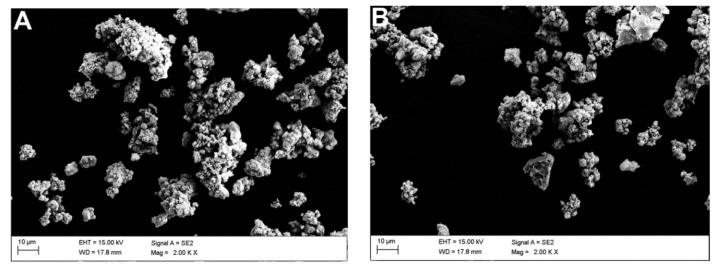
Morphology of the APTES-modified zirconia and alumina powders: (**A**) ZrO_2__250 and (**B**) ZrO_2__450.

**Figure 7:**
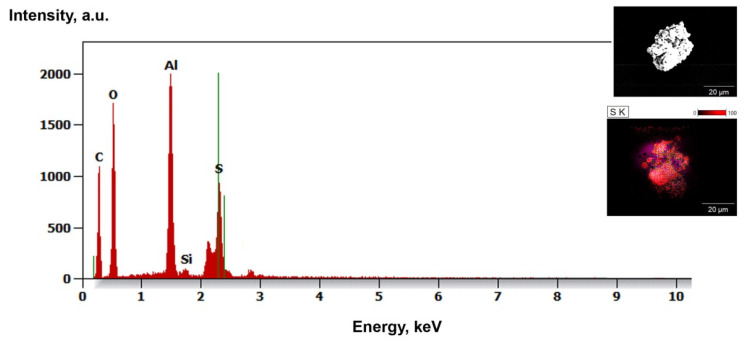
Sulphur contamination in modified alumina sample—Al_2_O_3__450.

This phenomenon was more pronounced for alumina than zirconia (Figure 5A,B). Upon increasing the temperature, there was an obvious thickening of the structure and the particles were more difficult to disintegrate (Figure 6A,B). In the case of alumina, a strong tendency to bind with sulphur from the etching process was observed (Figure 7).

**Figure 8:**
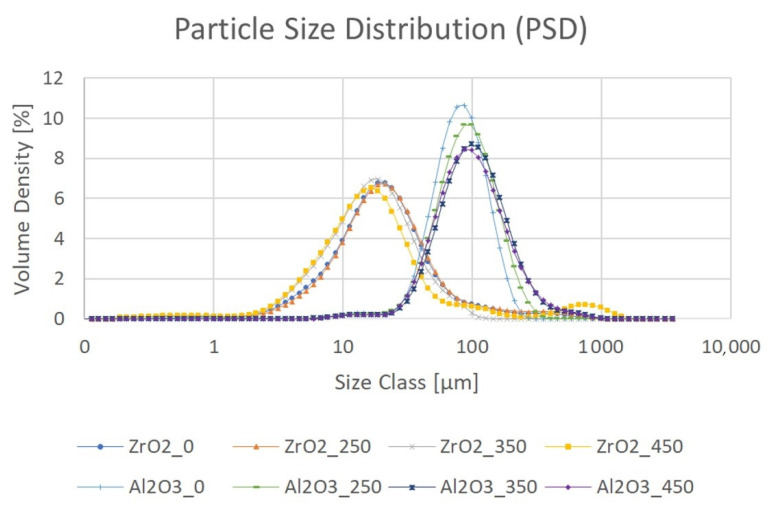
PSD of APTES-modified ZrO_2_ and Al_2_O_3_ powders.

The PSDs of the ZrO_2_ and Al_2_O_3_ modified powders are described in Figure 8 and Table 3 and Table 4. These results are the mean of five measurements. The PSD measurements were made after the last modification process, i.e., calcination. Only two samples, ZrO2_0 and Al2O3_0, were not calcined to provide a comparison. The ZrO_2_ powder had a larger PSD but smaller particles compared to the Al_2_O_3_ powder (Figure 8).

The PSD measurements of the powders after calcination at different temperatures showed that for ZrO_2_, the main peak did not vary in a particular direction, but between 100 and 2000 µm, the powder agglomeration became worse upon increasing the temperature. This could explain the high D90 value and standard deviation for the sample Al_2_O_3__450. For Al_2_O_3_, calcination flattened the curve and shifted it to the right, so both the PSD and agglomeration increased with temperature. Compared with ZrO_2_, agglomeration did not appear to be as severe because the curves dropped very steeply.

**Figure 9:**
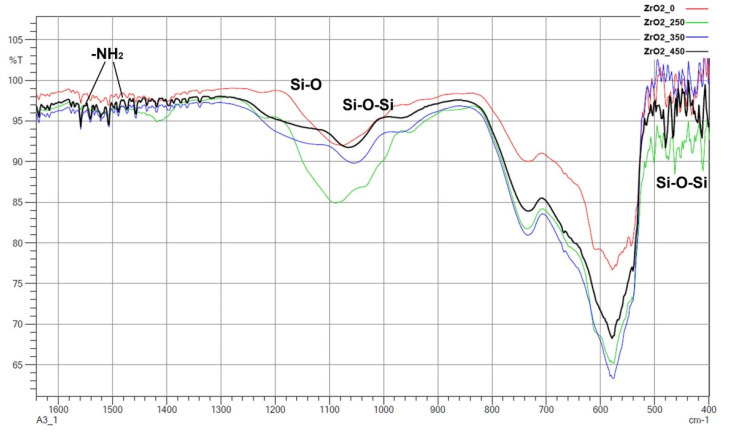
FTIR spectra of all APTES modfied zirconia samples.

**Figure 10:**
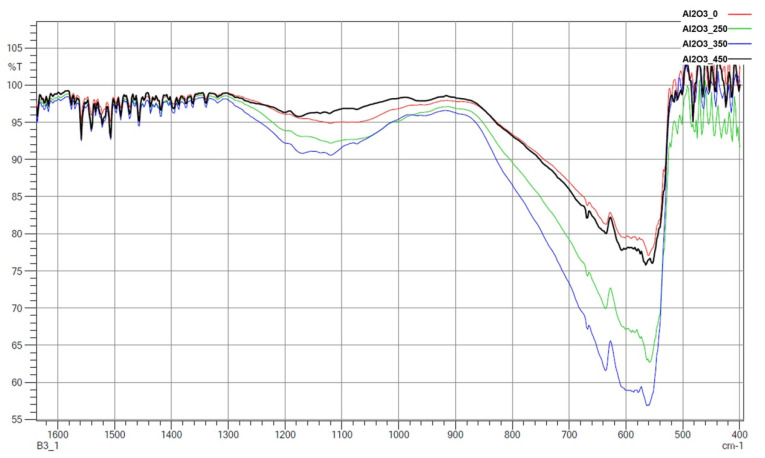
FTIR spectra of all APTES-modified alumina samples.

FTIR spectra are presented for zirconia (Figure 9) and alumina (Figure 10). Most importantly, -NH_2_ groups were present, which can improve the adhesion of the ceramic to the polymer. Two peaks responsible for -NH_2_ deformation modes at 1562 cm^−1^ and 1484 cm^−1^ were identified, which strongly confirm hydrogen-bonded structures with silanol groups to the zirconia and alumina [29,30,31,32]. There were also peaks at 1143–1199 cm^−1^, which were associated with the Si–O moieties of either polymerized or physisorbed APTES [31]. In the range of 1000–1100 cm^−1^, Si–O stretching/Si–O–Si stretching peaks were found [29,31]. The peak around 460 cm^−1^ was due to the absorption mode of Si–O–Si siloxane groups. These observations were the same for both zirconia and alumina. This seems to be in good correlation with the results obtained from EDS (Table 2) and seems to confirm that silanization has taken place.

**Figure 11:**
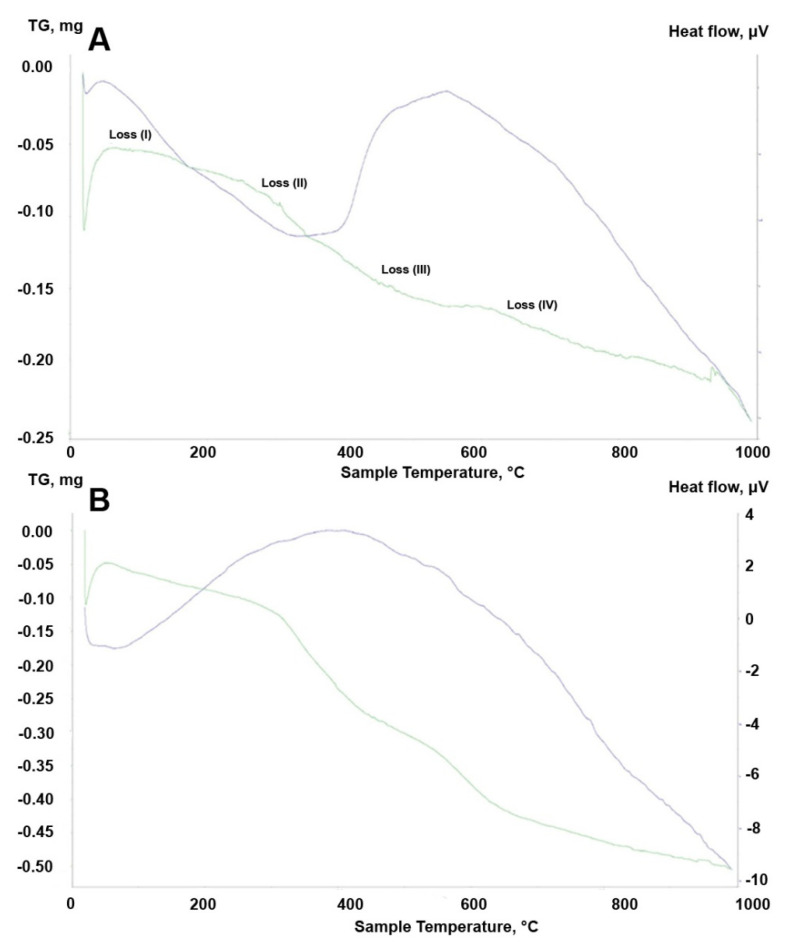
TGA curves of (**A**) ZrO_2__0 and (**B**) Al_2_O_3__0.

**Figure 12:**
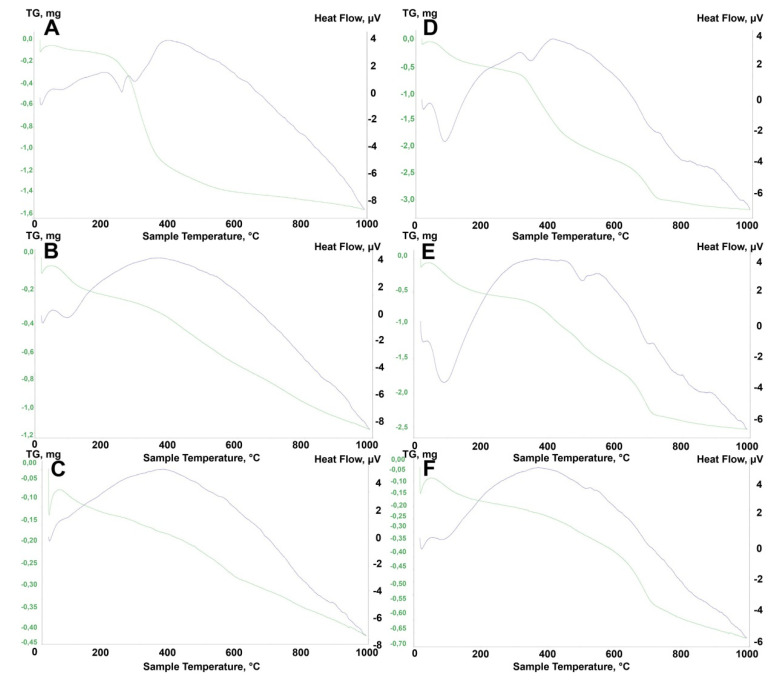
TGA curves of (**A**) ZrO2_250, (**B**) ZrO2_350, (**C**) ZrO2_450, (**D**) Al2O3_250, (**E**) Al2O3_350, and (**F**) Al2O3_450.

The TGA curves of the thermally-untreated samples (Figure 11A,B) and all thermally-treated samples are presented in Figure 12A–F. For both the ZrO2_0 and A2O3_0 samples (uncalcined), four characteristic areas of weight loss were observed (Figure 11A,B). For ZrO2_0, the following areas of mass loss were distinguished: weight loss (I) 70–160 °C, loss (II) 180–340 °C, loss (III) 360–530 °C, and loss (IV) 600–770 °C. Loss (I) is associated with physically-adsorbed water and the partial decomposition of APTES. Loss (II) was attributed to hydrogen-bonded APTES in the cross-linked framework. The loss (III) curve shows a multi-step thermal decomposition, during which the APTES grafted to the SiOH groups decomposed. In the final range above 500 °C, the dehydroxylation of residual ZrOH groups occured. Loss (IV) is associated with the dehydroxylation of grafted APTES species [32,33,34]. For alumina, the curve has a similar shape, but the thermal decomposition is more linear without several stages. For thermally treated zirconia powders (ZrO2_250, ZrO2_350, ZrO2_450), as expected, fewer characteristic areas of thermal decomposition were observed: loss (I) around 100 °C was related to the elimination of adsorbed water, and loss (II) around 400 °C was due to the one-step decomposition of APTES grafted to the SiOH groups and final dehydroxylation. For the ZrO2_250 sample, a deviation in the form of a sharp mass loss (220–400 °C) was noted. For the alumina Al2O3_0 sample, the same stages were observed as for ZrO2_0. (Figure 12B). For the remaining thermally treated samples, characteristic areas of mass loss were found, as for the zirconia samples. The difference was that no dehydroxylation of APTES-grafted species was observed (Figure 12D–F).

## 4. Discussion

It was found from XRD that the chemical modification process had no effect on the phase composition of either alumina or zirconia. In both cases, calcination did not change the phase composition, and α-alumina (rhombohedral, R-3c) and monoclinic zirconia (P21/c) were found in the XRD patterns (Figure 1 and Figure 2). The presence of silica compounds derived from the thermal decomposition of APTES was only observed in the XRD patterns of alumina. In the zirconia samples, they were obscured by the wider zirconia peaks. The EDS results confirmed the presence of silicon and nitrogen compounds in all samples—independent of the thermal treatment—for both zirconia and alumina samples (Table 2). Both elements were derived from the thermal decomposition of APTES [21]. The occurrence of silicon compounds in the alumina samples’ XRD patterns and EDS spectra was also confirmed by FTIR and TGA. In the FTIR spectra, amino groups and siloxane bonds were observed, regardless of the type of ceramic or calcination temperature. The non-calcined samples contained more intense peaks originating from the Si–O moieties of either polymerized or physisorbed APTES [31]. This trend was also observed for the peaks of -NH_2_ and the Si stretching of Si–O–Si groups (Figure 9 and Figure 10). The differences in spectral intensities between zirconia and alumina were due to the morphology of the powders. Zirconia has a smaller grain size and, therefore, a larger surface area development. Increasing the calcination temperature caused zirconia to clump into larger agglomerates, as confirmed by the PSD results (Table 3). These agglomerates formed compact structures with better densification ability during heat treatment (Figure 6); thus, they have a smaller available surface area to bind with compounds other than ceramics with a larger grain size and weaker intermolecular interactions, i.e., alumina. The modified alumina powders formed a looser structure with a lower compactability, making them more susceptible to the attachment of other compounds, which was also confirmed by the EDS results. This indicates the deposition of a large concentration of sulphur (Table 2). These conclusions were confirmed by comparing the EDS results of non-calcined ZrO_2__0 and Al_2_O_3__0 samples, which showed higher concentrations of N and Si for alumina than for zirconia (Table 2); however, as the calcination temperature and the density of the structure increased, the bonds between the compounds on the ceramic surface and the ceramic grains became more durable for zirconia, which was confirmed by the smaller decreases in the Si and N concentrations (Table 2). Weaker bonding was observed for alumina powders.

The TGA results were in fairly good agreement with the XRD, FTIR, and EDS observations. The most representative were the non-calcined ZrO_2__0 and Al_2_O_3__0 samples, in which the same four areas were distinguished during thermal analysis (Figure 11A,B). Based on the analysis of these two curves, it can be concluded that the thermal decomposition proceeded in a more complex way for ZrO_2__0, where several multipoint smaller decompositions were observed in individual stages. Relating these phenomena to the FTIR and EDS results, it can be concluded that the APTES-coated materials were more thermally stable in zirconia than in alumina. In the case of the calcined samples, the curves were more linear, and the correlations were more severe. For both zirconia and alumina, no dehydroxylation of the APTES-coated compounds was observed (Figure 12A–F).

## 5. Conclusions

The objective of this paper was to modify the surface of zirconia and alumina powders with 3-aminopropyltriethoxysilane (APTES) and determine the effect of selected calcination temperatures on the phase composition, morphology, and functional groups on the ceramic surface. For this purpose, a two-step modification process was developed: (1) etching in piranha solution and (2) direct silanization in dilute APTES in 2-propanol. The prepared samples were calcined at 250, 350, or 450 °C. The control group consisted of samples that did not undergo calcination. As a result of the analyses, the following relationships were obtained:

1. The developed process modified the surface of the selected powders. Silanization was carried out to introduce silane and amine groups on the surface, which was confirmed by XRD, EDS, FTIR, and TGA results.
2. The two-stage process did not affect the phase composition of the ceramic powders, while after the first etching in piranha solution, chemical impurities in the form of residual sulphur remained, which was more evident in the alumina powders.
3. Depending on the type of ceramics, differences were observed in the manner of surface modification. Zirconia bound less strongly to the introduced compounds, while thermal treatment had a weaker influence on the decrease in the concentration of the introduced compounds. This was completely different in the case of the alumina powders because zirconia showed a greater tendency to agglomerate and achieve a more compact structure during calcination. Alumina, on the other hand, showed a lower density and susceptibility to compacting of its structure during thermal treatment.
4. As expected, a thickening of the ceramic structure was observed upon increasing the temperature. The effect was more pronounced for zirconia than for alumina. At the same time, a decrease in the concentration of compounds due to the thermal decomposition of APTES was observed upon increasing the temperature. The effect was more pronounced for alumina than for zirconia. In addition, the TGA results correlate with the EDS results, as can be seen from the concentration of carbon in the samples, which may originate from the thermal decomposition of APTES. The carbon chains from the aminosilanes were sufficiently stable so that carbon residues were observed as a result of the decomposition, especially for samples that were calcined at 350 °C.
5. Future work should focus on adding an additional step to the process to neutralize the sulphur and introduce a subsequent step after thermal treatment to eliminate organic residues to homogenize the powders, e.g., grinding in a planetary or jet mill to eliminate subsequent media and drying.
6. At this stage of our investigations, we have established that a valuable composite can be made from the modified powders. However, in order to determine whether it will be suitable for medical applications, more thorough future studies need to be carried out, including determination of compliance with the requirements of standard 10993-1: “Biological evaluation of medical devices, simulation tests of artificial aging according to standards on aging of polymers for medical applications and printing of real models”, e.g., subjecting dental bridges to tests in a chewing simulator and analysis of tribological wear.

## Figures and Tables

**Table 1:** Description of etched, APTES-modified, and heat-treated samples.

Temperature, °C	Zirconia, -	Alumina, -
without thermal treatment	ZrO2_0	Al2O3_0
250	ZrO2_250	Al2O3_250
350	ZrO2_350	Al2O3_350
450	ZrO2_450	Al2O3_450

**Table 2:** Results of the chemical composition for all samples analysis with an EDS probe.

Sample	Concentration, wt.%						
	C	N	O	Si	S	Zr	Al
**ZrO_2_ (Raw)**	0	0	24.7	0	0	75.3	0
**ZrO_2__0**	7.45	2.50	18.45	1.075	0	70.75	0
**ZrO_2__250**	6.63	3.78	17.33	2.56	0.85	68.85	0
**ZrO_2__350**	9.6	3.53	18.55	5.65	0.33	62.34	0
**ZrO_2__450**	6.03	1.7	21.18	2.55	0	68.54	0
**Al_2_O_3_ (Raw)**	0	0	43.8	0	0	0	56.2
**Al_2_O_3__0**	6.05	3.7	38.7	1.93	11.45	0	38.17
**Al_2_O_3__250**	7.25	3.9	37.9	1.05	10.8	0	39.1
**Al_2_O_3__350**	19.00	2.00	29.68	0.67	9.35	0	39.3
**Al_2_O_3__450**	8.65	1.6	37.2	0.9	12.2	0	39.45

**Table 3:** PSD of the APTES-modified ZrO_2_ powders.

Sample	*D*10 (μm)	*D*50 (μm)	*D*90 (μm)
**ZrO2 (Raw)**	**4.1 (±0.08)**	**21.2 (±0.1)**	**48.63 (±0.74)**
**ZrO2_0**	6.49 (±0.07)	20.42 (±0.29)	61.42 (±6.76)
**ZrO2_250**	7.1 (±0.05)	21.36 (±0.29)	69.96 (±10.4)
**ZrO2_350**	5.76 (±0.02)	17.3 (±0.89)	43.14 (±0.85)
**ZrO2_450**	5.3 (±0.15)	16.76 (±0.85)	190.7 (±226.21)

**Table 4:** PSD of the APTES-modified Al_2_O_3_ powders.

Sample	*D*10 (μm)	*D*50 (μm)	*D*90 (μm)
**Al2O3_0 (Raw)**	**49.4 (±0.45)**	**94.5 (±0.22)**	**154.6 (±1.38**)
**Al2O3_0**	44.9 (±1.2)	85.06 (±0.77)	150.8 (±1.72)
**Al2O3_250**	47.74 (±0.05)	96.16 (±0.3)	182.2 (±1.17)
**Al2O3_350**	51 (±0.69)	107.6 (±1.02)	232.2 (±5.34)
**Al2O3_450**	48.12 (±0.23)	101.8 (±0.75)	231.8 (±5.23)

## Data Availability

The data presented in this study are available on request from the corresponding author.
